# Climate-driven changes of riparian plant functional types in permanent headwater streams. Implications for stream food webs

**DOI:** 10.1371/journal.pone.0199898

**Published:** 2018-06-28

**Authors:** María J. Salinas, J. Jesús Casas, Juan Rubio-Ríos, Enrique López-Carrique, José J. Ramos-Miras, Carlos Gil

**Affiliations:** 1 Department of Biology and Geology, University of Almería, Almería, Spain; 2 Andalusian Centre for the Evaluation and Monitoring of the Global Change (CAESCG), University of Almería, Almería, Spain; 3 Department of Education, University of Almería, Almería, Spain; 4 Department of Agronomy, University of Almería, Almería, Spain; Brandenburgische Technische Universitat Cottbus-Senftenberg, GERMANY

## Abstract

Little is known regarding consequences of climate change on riparian plant functional types (PFTs) related to leaf traits, with putative domino effects on stream food webs, plausible even if the tipping point of stream-desiccation is not reached. We hypothesized that, as stream food-webs are highly dependent on riparian subsidies, climate change might alter PFTs to the point of weakening terrestrial-aquatic linkages. We conducted a gradient analysis to assess the relative effects of climate, soil and riparian physical characteristics on PFTs. If PFTs differ significantly in leaf traits and climate had major influences on them, we could assume space-for-time interchangeability forward in time to predict leaf traits changes, and consequences for stream food webs under future climate change scenarios. Results indicated a clear distinction in leaf traits among PFTs: woody deciduous plants showed leaf traits associated to high decomposability and nutritional value for invertebrate shredders compared to evergreen woody and giant graminoid groups. We found a prime role of climate predicting changes in abundance and diversity of PFTs: 1) a warming and precipitation-decline scenario, coupled with soil characteristics related to aridification, would have detrimental effects on deciduous plants, while fostering giant graminoids; 2) in a scenario of no precipitation-reduction in wetter areas, warming might promote the expansion of evergreen to the detriment of deciduous plants. In both scenarios the net outcome implies increasing recalcitrance of leaf litter inputs, potentially weakening terrestrial-aquatic linkages in headwater streams.

## Introduction

The Earth system is suffering profound alterations due to human-caused global warming [1]. Although average precipitation is expected to increase at a global scale, for subtropical and mid-latitude regions future climate change scenarios predict increasing aridity and greater risk of heat waves [2]. This might have direct consequences on the hydrological cycle, such as decreasing stream discharge, severe reductions of summer flow, or desiccation [3]. In several Mediterranean areas, increasing aridity has already been detected over the past five decades [4] which, added to recurrent aridity events from the Upper Holocene, have caused major vegetation changes, i.e. reduction of forest cover and changes in species composition [5]. Understanding and predicting the consequences of ongoing climatic changes on forest ecosystems is a critical current challenge for scientists [6].

Headwater streams in forested temperate regions are fundamentally subsidized by organic detritus, particularly from riparian plants (e.g., [7]). Thus, changes affecting the quality and/or quantity of leaf litter inputs to streams may have the potential to alter nutrient transformation rates and fluxes [8, 9], and the trophic structure [10] and diversity [11] in these ecosystems.

As aridity increases, riparian ecosystems are subjected to decreased surface flow and increased depth of water table, determining declines in stream segments with perennial vs. intermittent/ephemeral, stream flow [12]. This leads to changes in plant community structure of riparian ecosystems [12, 13], which might significantly alter stream food webs [14]. There is, however, much less information available on the effects of aridity on riparian plant communities, and its implications for stream food webs, when this factor does not suppress surface flow.

In terrestrial ecosystems, a complex interplay among rainfall and temperature, in combination with climatic and soil factors, exert key effects on intraspecific phenotypic changes affecting leaf traits, determining species abundance and distribution if these factors exceed their tolerance threshold [15]. In perennial species, increasing aridity, often coupled with low soil nutrient availability, tends to favor traits that improve leaf-level water use efficiency and leaf lifespan, i.e. leaves with thick cuticles, and small, thick-walled cells [16]. These features lead to the generally low specific leaf area (SLA) and high toughness (sclerophylly) often reported from many drylands species [16, 17], which, in turn, slow down litter decomposability and digestibility [18].

Studies on riparian trees indicate notable intraspecific variability in leaf traits, affecting their decomposability, which is related implicitly or explicitly to climate and/or soil fertility [19, 20]. Effects of these factors on interspecific variability in riparian leaf traits from permanent low order streams remain almost unexplored (but see [21] for the effects of climate). In these ecosystems, the continuous accessibility to water by the root system, the relatively high rates of soil organic matter mineralization, and the influence of geomorphological characteristics of the valley hillsides and channel, besides periodic flood events causing alluviation pulses and disturbance on species population dynamics (e.g., [22]), are factors that might obscure direct effects of climate or soil characteristics on the quality/quantity of riparian-derived detritus entering streams.

Plant functional types (PFTs) classify species sharing similar functional traits, thus with similar responses to environmental gradients [17, 23]. This approach is therefore highly suitable for large-scale models of vegetation dynamics in a changing environment [18], including riparian ecosystems [24, 25]. Further, studies from diverse ecosystems worldwide indicate that PFTs -related to growth form (grass, herb, shrub, or tree) and leaf phenology (deciduous vs. evergreen)- are useful to predict leaf litter decomposability [18].

We adopted a ‘space-for-time substitution’ approach [26] to investigate potential climate-change effects on riparian PFTs in permanent low-order streams. We extrapolated their possible temporal dynamics from the spatial variation observed across a broad aridity gradient in near pristine sites from a relatively small region. The assumption of space-for-time substitutability, through the study of climatic gradients, has been shown to be quite satisfactory to model community responses to climate change (e.g., [27]), and it is being increasingly applied in climate change research in terrestrial [28] and aquatic [29] ecosystems.

We hypothesized (i) that since PFTs (defined in terms of growth forms and leaf phenology) differ in nutrient uptake and allocation strategies, they should differ fundamentally in key leaf traits related to litter decomposability, i.e. we expect a relationship between functional response traits (nutrient uptake and allocation strategies) and effect traits (leaf decomposability); (ii) that, given the high leaf-level responsiveness to climate [30], climatic variables should exert capital filter effects on abundance and diversity of PFTs; and (iii) that noticeable effects of soil and stream/riparian physical variables on PFTs could be expected, considering the large variation in geology across the studied climatic gradient. Ultimately, this study can contribute to the classical question in functional ecology of the relationship between response and effect traits [31].

## Material and methods

### Study area and stream selection

This study was conducted in Andalusia (Southern Spain) (38°43’N-36°00’N latitude; 07°31’W-01°37’W longitude) covering an area of ca. 88,000 km^2^ (S1 Fig). This region boasts considerable climatic, lithological and topographical heterogeneity. Within the context of Mediterranean-type climate, three main climate subtypes can be differentiated. *Mediterranean inland and mountainous climate* affects most of the region. *Mediterranean subtropical climate* occurs along most Mediterranean coastal zones. This is characterized by mild winter temperatures, with very variable precipitation, decreasing in a west-east gradient (1500–400 mm/year). *Mediterranean subdesert climate* is characteristic of the southeastern sector of the region, with annual precipitation lower than 200 mm in some areas. This region represents a complex mosaic of soils derived from marls, clays, limestones, gypsum, calcareous and siliceous sandstones, schists and phyllites.

All sites selected were first to second order streams, had perennial flow and at least a 100-m long accessible stream segment. They were located in protected areas (Nature Preserves, Natural or National Parks) to ensure high naturalness according to the standards for a Mediterranean region (e.g. see European Commission; wild areas in Natura 2000). Permissions for field studies were issued by the RENPA (Red de Espacios Protegidos de Andalucía). A total of 34 streams were selected (S1 Fig, S1 Table), most (24 streams) were located within the dominant *Mediterranean inland and mountainous climate*, and a smaller number were within the other two climate types (5 streams each). Thus, the climatic gradient studied (mean annual temperature range 10–18 °C; mean annual precipitation range 297–1,414 mm; Table 1) embraced the climate change projections for the Mediterranean region of warming (mean temperature rise 2–4 °C) and decrease in mean annual precipitation (between 10–40%) towards the end of the 21st century (reviewed by [2]). These climate change projections, for different models and scenarios, are essentially in line with those made for Andalusia (average temperature rise 2–4 °C; average precipitation reduction 14–27%) (REDIAM, http://www.juntadeandalucia.es/medioambiente/site/web/rediam).

**Table 1:** Environmental characterization of surveyed streams.

Environmental variables	Mean	SD	Min	Max
**Climate**				
Mean annual precipitation (mm) (PP)	833	325	297	1,414
Potential evapotranspiration (mm/y) (PET)	741	65	633	920
Number of days per year with precipitation (DaysPP)	58	17	28	89
Mean annual temperature (°C) (Tmean)	13.8	2.0	10.3	18.3
Mean minimum temperature of the coldest month (°C) (Tmin jan)	2.6	2.7	-1.3	8.2
Mean maximum temperature of the warmest month (°C) (Tmax jul)	29.5	2.3	22.9	33.0
Annual temperature range (°C) (Trange)	26.9	3.8	14.9	31.5
PP/PET	1.13	0.42	0.32	1.85
De Martonne aridity index	33	12	10	54
Emberger’s bioclimatic coefficient (Q2)	113	58	39	260
**Soil**				
Electric conductivity (μS cm^-1^) (EC)	1020	717	301	3290
pH_w_	7.37	0.96	5.25	8.81
% Calcium carbonate (%CaCO_3_)	18.97	27.38	0.01	85.86
% Organic carbon (%OC)	2.64	1.79	0.10	5.41
% Total nitrogen (%N)	0.122	0.089	0.017	0.358
Carbon to nitrogen ratio (C:N)	23.68	15.72	4.40	79.76
Extractable phosphorus (ppm) (P)	329	164	92	783
Cation exchange capacity (cmol^+^ kg^-1^) (CEC)	14.89	9.69	2.68	47.80
% Available water (%AW)	4.79	2.26	1.26	11.07
% Base saturation (%BS)	88.88	15.77	45.69	100.00
Exchangeable sodium % (ESP)	2.65	5.71	0.13	32.39
**Physical**				
Altitude (m asl)	763	391	47	1,465
Slope (%)	20	5	9	31
Drained-basin area (ha)	559	346	12	1,480
Valley southern orientation (degrees)	67	65	1	180
Bankfull/floodplain channel width (m)	9	5	3	19
Active wetted channel width (m)	2.2	1	0.75	5
% Hard stony substrate (%Hard substrate)	34	23	5	80
Discharge (L s^-1^)	98	147	3	597

Mean, standard deviation (SD) and range values of environmental variables measured for 34 low-order streams from southern Spain, used as predictor variables of plant functional types in PLS regression models.

### Climatic and physical characteristics at basin and local scales

We used the Zonal Statistics tool, (Spatial Analyst Extension of ArcGIS 9.3, ESRI), to obtain average values of topographic and climatic variables from each stream reach and its watershed. All raster layers are available online at the website of the Environmental Information Network of Andalusia (REDIAM, http://www.juntadeandalucia.es/medioambiente/site/web/rediam). Climatic variables were obtained from raster layers with a pixel resolution of 100x100m. These layers contain annual average, maximum (warmest month) and minimum (coldest month) values of temperature, annual precipitation (PP), number of days per year with precipitation (Days PP), and potential evapotranspiration (PET), recorded along the last period with available climatic data (1971–2000) (Table 1). From these variables we calculated three phytoclimatic indices of aridity: the UNEP aridity index (PP/PET), the de Martonne’s aridity index, and the Emberger’s bioclimatic coefficient (Q2) [32]. Altitude (sampling site), average basin slope (%), drainage-basin area and valley orientation (0°, full northern orientation; 180°, full southern orientation) were obtained from raster format layers with a pixel resolution of 20x20m (Table 1). Other local physical characteristics were measured *in situ* along a 100 m stream reach during 2013–2014: average width of bankfull/floodplain and active wetted channels, % hard stony substrate along the riparian habitat, and stream discharge (Table 1). Measurements of discharge (and active wetted channel) of streams were taken *in situ* twice during the low (summer) and high (winter) flow periods over the two years’ study (total of four measurements per stream).

### Soil characteristics

At each 100 m stream reach, during June-July 2013, we collected a soil sample from the riparian soil, compounded by six core sampling units of the top 20 cm of the mineral soil profile. Sampling units from each site were taken by a randomly stratified (both stream sides) method, and were mixed, air dried, sieved (2 mm) and stored in sealed polyethylene bags until analyzed. Soil physical and chemical variables (Table 1) were measured as in [33]. Soil electric conductivity (EC) and pHw were measured in a 1:5 soil:water extract. Calcium carbonate content (%CaCO_3_) was measured by treatment with hydrochloric acid and quantification of evolved CO_2_ by gas volumetry. Soil organic carbon (%OC) was determined by wet digestion with potassium dichromate by the Walkley-Black method. Percent total N (N) was determined by Kjeldahl digestion. Extractable P (P) was determined by the Olsen’s method. Cation exchange capacity (CEC) was measured by saturating the sample with sodium acetate solution (pH = 7.0), then by replacement of sodium with ammonium acetate 1 M (pH = 8.2) and measurement by flame atomic absorption spectrometry (FAAS). Concentrations of exchanged Na and K were measured in the ammonium acetate extract by FAAS, and those of Ca and Mg were determined by AAS. CEC and exchangeable cations were used to calculate percent base saturation (%BS) and exchangeable sodium % (ESP). Percent available water (%AW) was obtained as the difference in soil humidity between two suctions (33 and 1500 KPa).

### Plant functional types and leaf traits

In each stream, vegetation data were collected between June-July 2013, in six plots (36 m^2^ each) randomly distributed in two strata -both stream sides, three plots per side- along a 100 m stream reach. Each quadrilateral plot was arranged from the edge of the wetted channel. We recorded percent coverage of all woody species and giant herbs (graminoids and forbs), the most conspicuous plant species that could produce significant leaf litter inputs to streams. Species were identified by [34] and [35], and their cover was estimated by the Domin-Krajina scale of cover and abundance [36]. Plant species were categorized into six functional types according to their growth form (graminoid, forb, shrub, tree) and leaf phenology (deciduous vs. evergreen): giant graminoids, giant forbs, evergreen shrubs, deciduous shrubs, evergreen trees, and deciduous trees. The type evergreen tree was dominated by broad leaves species, with conifers being less abundant. As forbs were not as frequent nor abundant as expected, we did not include it in the analyses.

At the same 100 m stream reach, total canopy cover was estimated using 10 vertical photographs taken in each sampling site (at 10 m intervals) with a wide-angle lens in summer foliage. We adjusted the contrast of photographs to make open sky pure white and solid objects pure black using Adobe Photoshop v7.0 (Adobe Systems Inc.).

We measured specific leaf area (SLA), toughness, C, N, lignin and Si as proxies of leaf decomposability (e.g., [37]) and nutritive value for detritivores [38]. These measurements were carried out for the most frequent and abundant species: giant graminoids (n = 3), evergreen shrubs (n = 6) evergreen trees (n = 5), deciduous shrubs (n = 3) and deciduous trees (n = 15) (see S1 Appendix for species identity). Leaves of each species were collected (June-July 2013) from a number of streams ranging between 3 and 33, depending on how frequent and abundant the species was. Methods followed the recommendations of [39] unless otherwise stated. For each species at a given site, we collected 102 leaves from 6 individuals (17 leaves per individual). Leaves were stored dry in the dark at room temperature until processed. Before measurements, leaves were rehydrated by spraying with distilled water and stored for 12 h at 5 °C. SLA (mm^2^ g^-1^) was determined by measuring area, using the WinDIAS 3 Leaf Area Meter System (Delta-T devices), and dry mass (60 °C, 78 h) of individual leaves. Leaf toughness was measured (distal and proximal punctures, per individual leaf) using a calibrated texturometer (TA.XT2 Plus, Stable Micro Systems). A constant needle tip surface area (0.38 mm^2^) was used throughout all measurements, thus differences in toughness were expressed in units of mass (g). C and N content of leaves was determined using a mass spectrometer (EA-Thermo DELTA V Advantage, Fisher Scientific^®^), and expressed as %C_mass_ and %N_mass_ of leaf dry mass. Concentration of lignin was estimated gravimetrically using the acid-detergent method of [40]. The concentration of silicon (Si) was measured using inductively coupled plasma atomic emission spectroscopy (Thermo ICAP 6500 duo, Fisher Scientific^®^), after microwave digestion in nitric acid (65%) and hydrogen peroxide (30%).

### Statistical analyses

We compared mean leaf SLA, toughness, %N_mass_, C:N, %lignin and Si among the five PFTs using one-way ANOVAs, followed by Tukey HSD *post hoc* tests (unequal sample sizes) for pair wise comparisons.

We determined the natural number of clusters (range 2–5), i.e. number of species groups showing similar leaf traits, to investigate the congruence of the five PFTs a priori differentiated. To do so, we used the Elbow method, using K-means clustering. Prior to ANOVAs and cluster analyses, dependent variables were log-transformed.

We used partial least squares regression (PLS) to evaluate the relative importance of climate, soil and physical variables (Table 1) as predictors of %cover and diversity of PFTs. PLS regression extracts, from a set of independent variables, orthogonal “latent” components which are then used as independent variables in regression, thus circumventing problems of multicollinearity among a high number of predictors. This approach is more reliable than other techniques when identifying relevant variables and their magnitudes of influence, especially in cases of small sample size [41].

We first conducted separate PLS regression analyses for %cover and Simpson diversity of the five functional types as dependent variables (multivariate dependent variable models), but using in both cases three matrices of predictors-climate, soil and physical-and their four possible combinations. This was intended to evaluate the predictive power of each matrix, and how this was modified by the presence of the other matrices [42]. Correlation analyses among predictors within each matrix, and a preliminary PLS regression, were used to reduce the number of variables, in order to equal the size of matrices to 8 independent variables per matrix. Thus, PET, de Martonne aridity index, and soil N (r > 0.85 with PP/PET, Emberger’s bioclimatic coefficient (Q2) and C:N, respectively), and %base saturation and % available water (%AW) -with the lowest variable importance in projection (VIP) (see below) among soil variables- were removed from definitive PLS analyses.

Secondly, we ran separate PLS regression analyses for each PFT (univariate dependent variable models) using in this case a unique matrix of predictors (the sum of the three matrices above). This was intended to evaluate differences in predictive power of PLS models among PFTs and the relative importance of individual independent variables as predictors of %cover and diversity for each PFT, %cover total canopy and total diversity. The relative influence of each predictor variable in the model was expressed as the variable importance in the projection (VIP). The PLS-VIP method performs excellently in identifying relevant predictors and outperformed other methods: predictors with VIP values > 1 are considered the most relevant [43]. Prior to definitive PLS analyses, we used VIP as a criterion for variable selection to improve model prediction and interpretation [43]. Thus, only the 15 independent variables with VIP > 0.9 in a preliminary analysis, for each PFT separately, were included in the final analysis.

In multivariate and univariate dependent PLS regressions, the number of extracted components and the robustness of the resulting models were determined by leave-N-out cross-validation (LNO). Samples were randomly divided into five groups (each group representing ~20% of the total set of samples). Five models were developed with one of the sample groups omitted (training set). For each model, we determined the optimum number of latent components using the option maximum Q^2^ (the proportion of variance in the response variable that can be predicted by the model), in which new components were added to the model if they were statistically significant (Q^2^ > 0.097, *P* < 0.05) [39]. We also determined R^2^ (Y), the proportion of variance in the response variable that was explained by the model. Mode and range of N (number of significant latent components), mean and standard deviation of Q^2^ and R^2^ (Y), of the 5 models resulting from the LNO cross-validation process, were given as indicator of the statistical robustness of PLS models.

Prior to calculations, predictor variables were centered and scaled to unit variance to give all variables the same relative importance. Response variables of %cover required arcsine-square root transformation to minimize deviations from normality.

Statistical analyses were carried out using XLSTAT version 2014.4.10 (Addinsoft, Inc.).

## Results

### Vegetation types, plant functional types and leaf traits

Five main types of riparian vegetation, according to EUNIS habitat classification (http://eunis.eea.europa.eu), were recorded: *Alnus* galleries, Iberian poplar galleries, Iberian meso-Mediterranean ash galleries, oleander galleries, and willow formations (scrubs, galleries or woods) (S1 Table).

**Fig 1:**
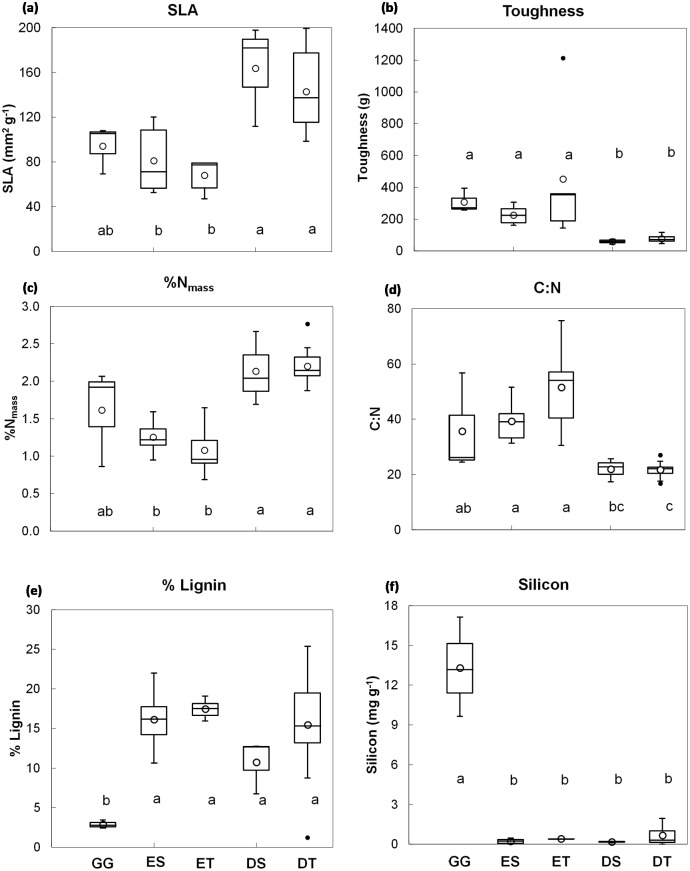
Box-and-whisker plots for selected leaf trait variables of the five plant functional types studied. Giant graminoids (GG), evergreen shrubs (ES), evergreen trees (ET), deciduous shrubs (DS) and deciduous trees (DT). Box represents median and 25th and 75th percentile levels, open dot is the mean, whisker is the range, and closed dots are outliers. Different letter indicates significant (P < 0.05) differences among plant functional types.

Plant functional types differed significantly in SLA, toughness, %N_mass_, C:N ratio, %lignin and Si (one-way ANOVAs: *F*_4, 31_ = 11.2, *P* < 0.001; *F*_4, 31_ = 23.7, *P* < 0.001; *F*_4, 31_ = 16.5, *P* < 0.001; *F*_4, 31_ = 16.9, *P* < 0.001; *F*_4, 31_ = 6.3, *P* < 0.01; *F*_4, 31_ = 39.2, *P* < 0.0001; respectively) (Fig 1). Deciduous (shrubs and trees) showed significantly higher SLA and %N_mass_, but lower toughness and C:N ratio, compared to evergreen plants (Tukey HSD, *P* < 0.001, in all pair wise comparisons), but these did not differ significantly in lignin and Si concentration (Fig 1). Giant graminoids (GG) showed significantly higher concentration of Si, but lower %lignin, than other groups; and toughness and C:N ratio were significantly higher in GG compared to both deciduous groups and to deciduous trees, respectively (Tukey HSD, *P* < 0.001) (Fig 1).

K-means clustering indicated that the best solution was that shaped by 3 species groups—giant graminoids (GG), evergreen (ET+ES) and deciduous (DT+DS) species—since a clear elbow was observed at K = 3 (intra-group variance = 30%; inter-group variance = 70%), and all species were correctly classified, except one DT species (*Ficus carica* L.) that was clustered within GG.

### Relative importance of the three environmental matrices and their combinations as predictors of plant functional types

Multivariate dependent variable models showed how the three evaluated matrices of independent variables, and their combinations, were able to predict and explain the variation of %cover and diversity of PFTs (Table 2; S2 Fig for biplots of correlations). Models using climate variables as predictors were the most parsimonious (predictive power always significant; Q^2^ > 0.097), and variance explained of response variables was higher compared to models using soil or physical variables alone. This was particularly true when predicting diversity, because only models including climate variables were significant. More significant, robust and stable models (higher Q^2^ and R^2^ means and lower standard deviations in LNO cross-validation of models; Table 3) were found when predicting %cover of PFTs from climate factors or their combinations with other environmental factors. Adding soil to climate variables increased slightly robustness and stability (Table 2) of models predicting %cover, but decreased stability of predictive power (higher standard deviations of Q^2^ in LNO cross-validation of models). Adding physical predictors produced worse models (Table 2).

**Table 2:** Summary of multivariate dependent variable PLS models fitted to %cover and diversity of plant functional types.

Dependent variable and set of environmental variables or combinations	Full model			Leave-N-out cross-validation of model				
	N	Q^2^	R^2^(Y)	N	Q^2^_LNO_		R^2^_LNO_ (Y)	
				mode (range)	mean	SD	mean	SD
*%cover*								
**Climate (C)**	**2**	**0.239**	**0.368**	**2 (2–2)**	**0.213**	**0.042**	**0.374**	**0.028**
**Soil (S)**	**1**	**0.184**	**0.262**	**1 (1–2)**	**0.158**	**0.079**	**0.285**	**0.071**
Physical (P)	1	-0.040	0.111	1 (1–2)	-0.035	0.088	0.204	0.077
**C+S**	**2**	**0.275**	**0.412**	**2 (2–2)**	**0.239**	**0.058**	**0.418**	**0.027**
**C+P**	**3**	**0.237**	**0.467**	**2 (2–3)**	**0.203**	**0.049**	**0.449**	**0.047**
**S+P**	**2**	**0.160**	**0.395**	**2 (2–4)**	**0.137**	**0.070**	**0.421**	**0.086**
**C+S+P**	**2**	**0.271**	**0.433**	**2 (2–3)**	**0.226**	**0.058**	**0.457**	**0.028**
*Simpson diversity (1-D)*								
**Climate (C)**	**3**	**0.149**	**0.327**	**3 (2–3)**	**0.113**	**0.062**	**0.319**	**0.040**
Soil (S)	3	0.052	0.295	1 (1–3)	0.006	0.063	0.177	0.112
Physical (P)	1	0.002	0.102	1 (1–1)	-0.028	0.061	0.111	0.013
**C+S**	**3**	**0.146**	**0.368**	**3 (1–3)**	**0.103**	**0.071**	**0.334**	**0.111**
**C+P**	**3**	**0.137**	**0.361**	**2 (1–3)**	**0.099**	**0.064**	**0.316**	**0.109**
S+P	3	-0.005	0.357	1 (1–3)	-0.018	0.050	0.210	0.121
**C+S+P**	**3**	**0.122**	**0.390**	**3 (1–3)**	**0.088**	**0.074**	**0.346**	**0.120**

The number (mode and range) of significant PLS components (N), the proportion (mean and standard deviation, SD) of the variance in the response variables that can be predicted by the model (Q^2^), and the coefficient of determination (mean and standard deviation) (R^2^) are shown for each model. Significant models (Q^2^ > 0.097, P < 0.05) are in bold.

**Table 3:** Summary of univariate dependent variable PLS models fitted to %cover and diversity of plant functional types.

%cover or diversity of plant functional types	Full model			Leave-N-out model cross-validation				
	N	Q^2^	R^2^(Y)	N	Q^2^_LNO_		R^2^_LNO_(Y)	
				mode (range)	mean	SD	mean	SD
*%cover*								
**Giant graminoids**	**1**	**0.685**	**0.752**	**1 (1–1)**	**0.656**	**0.054**	**0.750**	**0.033**
**Evergreen shrubs**	**1**	**0.413**	**0.513**	**1 (1–1)**	**0.415**	**0.085**	**0.533**	**0.075**
**Evergreen trees**	**1**	**0.221**	**0.367**	**1 (1–1)**	**0.224**	**0.083**	**0.399**	**0.068**
Deciduous shrubs	1	-0.930	0.249	1 (1–1)	-0.126	0.065	0.280	0.057
**Deciduous trees**	**1**	**0.477**	**0.604**	**1 (1–1)**	**0.443**	**0.080**	**0.610**	**0.041**
**Total canopy cover**	**1**	**0.372**	**0.533**	**1 (1–1)**	**0.395**	**0.132**	**0.607**	**0.139**
*Simpson diversity (1-D)*								
**Giant graminoids**	**1**	**0.511**	**0.578**	**1 (1–1)**	**0.499**	**0.049**	**0.587**	**0.031**
**Evergreen shrubs**	**1**	**0.245**	**0.349**	**1 (1–1)**	**0.230**	**0.059**	**0.362**	**0.037**
**Evergreen trees**	**1**	**0.339**	**0.452**	**1 (1–1)**	**0.298**	**0.160**	**0.458**	**0.098**
**Deciduous shrubs**	**1**	**0.240**	**0.424**	**1 (1–1)**	**0.249**	**0.109**	**0.467**	**0.079**
**Deciduous trees**	**1**	**0.253**	**0.410**	**1 (1–1)**	**0.225**	**0.127**	**0.424**	**0.067**
Total diversity	1	0.102	0.268	1 (1–1)	0.082	0.093	0.304	0.053

The number (mode and range) of significant PLS components (N), the proportion (mean and standard deviation, SD) of the variance in the response variables that can be predicted by the model (Q^2^), and the coefficient of determination (mean and standard deviation) (R^2^) are shown for each model. Significant models (Q^2^ > 0.097, P < 0.05) are in bold.

### Environmental determinants and predictive power for individual plant functional types

Models predicting %cover and diversity of individual PFTs (univariate dependent variable models) from the 15 selected predictors in the three matrices (climate+soil+physical) were significant, except for %cover of deciduous shrubs and total diversity (Table 3). In all models the optimum number of latent components retained was one, with no variation in LNO cross-validation runs. Models for %cover always showed higher predictive power and explained higher proportion of variance in the response variable compared to models for diversity, except in deciduous shrubs. Model stability was higher (low standard deviation of Q^2^ and/or R^2^ in LNO cross-validation runs) for diversity, compared to %cover, of giant graminoids and evergreen shrubs, but the reverse pattern was obtained for evergreen and deciduous trees.

Giant graminoids (GG) showed the highest predicted (Q^2^ in full and LNO models) and explained (R^2^ in full and LNO models) variance fractions of response variable, and the highest model stability (Table 3). Environmental predictors with the highest influence (VIP >1), both on %cover and diversity of GG, were proxies of aridity (positive effects from low precipitation and high temperature) and associated soil properties: positive effects from high EC, pH_w_, exchangeable sodium % and % base saturation, but low %OC (Tables 4 and 5). Moreover, the gap in VIP scores between pH_w_ (1.516) and other important climate and soil predictors (starting with mean temperature VIP = 1.192) of GG diversity, highlights the importance of pH_w_ in this model.

**Table 4:** Variable importance in the projection (VIP) and standardized coefficients of the variables used in the PLS models with %cover of each plant functional type as dependent variable and 15 selected variables from the three matrices (climate, soil, physical) as explanatory variables.

Dependent variable	Predictor variable	VIP		Standardized coefficient	
		mean	SD	mean	SD
Giant Graminoids	EC	1.365	0.391	0.159	0.045
	PP/PET	1.239	0.189	-0.144	0.024
	Annual mean temperature	1.199	0.231	0.140	0.033
	pH_w_	1.178	0.244	0.137	0.035
	ESP	1.173	0.478	0.137	0.056
	PP	1.041	0.217	-0.121	0.024
Evergreen shrubs	PP	1.429	0.176	0.114	0.017
	Q2	1.366	0.137	0.109	0.016
	PP/PET	1.309	0.229	0.105	0.020
	Min temperature January	1.231	0.213	0.098	0.021
	Altitude	1.158	0.320	-0.092	0.025
Evergreen trees	Days PP	1.472	0.361	0.118	0.039
	PP/PET	1.422	0.305	0.114	0.031
	PP	1.241	0.339	0.099	0.032
	Q2	1.092	0.398	0.086	0.033
	Active channel width	1.088	0.513	0.087	0.047
Deciduous trees	EC	1.552	0.344	-0.160	0.045
	ESP	1.546	0.311	-0.159	0.038
	Annual mean temperature	1.415	0.250	-0.146	0.032
	Altitude	1.302	0.271	0.134	0.031
	% Hard substrate	1.114	0.413	-0.114	0.038
% Total canopy cover	pH_w_	1.394	0.212	-0.135	0.042
	PP/PET	1.233	0.254	0.102	0.036
	%Base saturation	1.214	0.307	-0.125	0.051
	PP	1.194	0.244	0.098	0.035
	Q2	1.167	0.210	0.092	0.033
	%OC	1.095	0.364	0.099	0.052
	EC	1.074	0.570	-0.117	0.063

Only the most influencing variables (VIP > 1) are shown.

**Table 5:** Variable importance in the projection (VIP) and standardized coefficients of the variables used in the PLS models with diversity of each plant functional type as dependent variable and 15 selected variables from the three matrices (climate, soil, physical) as explanatory variables.

Dependent variable	Predictor variable	VIP		Standardized coefficient	
		mean	SD	mean	SD
Giant Gramminoids	pH_w_	1.516	0.224	0.149	0.031
	Annual mean temperature	1.192	0.268	0.117	0.029
	%OC	1.187	0.245	-0.117	0.026
	PP/PET	1.153	0.224	-0.113	0.023
	% Base saturation	1.012	0.214	0.100	0.025
	Days PP	1.002	0.300	-0.098	0.030
Evergreen shrubs	% Hard substrate	1.446	0.393	0.094	0.028
	Q2	1.328	0.172	0.086	0.017
	Min temperature January	1.215	0.286	0.079	0.021
	PP	1.215	0.270	0.079	0.024
	PP/PET	1.112	0.290	0.072	0.024
	Altitude	1.103	0.375	-0.071	0.027
	Days PP	1.027	0.339	0.066	0.027
Evergreen trees	Annual temperature range	1.674	0.287	-0.124	0.034
	Min temperature January	1.466	0.205	0.108	0.020
	Q2	1.433	0.278	0.106	0.037
	Max temperature July	1.017	0.512	-0.076	0.057
Deciduous shrubs	Days PP	1.417	0.476	0.146	0.058
	C:N	1.345	0.450	0.140	0.055
	Temperature annual range	1.336	0.374	0.138	0.041
	Max temperature July	1.210	0.381	0.126	0.047
	%CaCO3	1.097	0.494	0.112	0.049
Deciduous trees	Altitude	1.296	0.377	0.118	0.036
	%OC	1.259	0.534	0.115	0.054
	Annual mean temperature	1.221	0.413	-0.112	0.040
	P	1.094	0.529	0.099	0.051
	Valley southern orientation	1.043	0.632	-0.096	0.065
	ESP	1.026	0.537	-0.095	0.052
	%CaCO_3_	1.003	0.521	0.093	0.047

Only the most influencing variables (VIP > 1) are shown.

The model for %cover of deciduous trees (DT) was the second best in predictive/explanatory power and stability (Table 3). This represents a gradient of aridity similar, but with inverse effects to that obtained for GG, with prime negative effect of soil factors—EC and exchangeable sodium %—followed by mean temperature; although altitude, as well as to a lesser extent % hard stony substrate, were also important predictors (Table 4).

Diversity of DT was predicted by a more complex model, where the most important predictors were altitude and %OC -with positive effects- and mean temperature with negative effect; less importantly, other predictors contributed with positive (%CaCO_3_ and extractable P) or negative effects (valley southern orientation and exchangeable sodium %) (Table 5).

No significant model was obtained for %cover of deciduous shrubs (DS) but a significant and relatively stable model, particularly for R^2^ (with low SD in LNO cross-validation runs), was obtained for its diversity (Table 3). This model selected 3 climate and 2 soil variables as important predictors (Table 5), which, overall, suggests that diversity in this group increases towards headwater streams over calcareous substrates under humid and continental climate.

The %cover and diversity of evergreen -shrubs and trees- were primarily predicted by climate variables, without major contribution of soil factors. Generally, abundance and diversity of these groups were favored by precipitation and mild winters (Tables 4 and 5), but, apart from these similarities, several differences can be pointed out. The principal predictor with positive effect on diversity of evergreen shrubs was % stony hard substrate. This suggests an indirect positive effect of this factor on shrubs diversity mediated by an open tree canopy, since hard substrate showed noteworthy negative effect on %cover of deciduous trees (Table 4). Moreover, annual range of temperature and maximum temperature of July showed important negative effects on evergreen trees diversity (Table 5).

A significant model predicting %cover of total canopy explained a relatively high proportion of variance (R^2^ = 0.61) in the dependent variable, but with lower predictive power and stability compared to most individual PFTs (Table 3). As could be expected, the set of important predictors of this variable was similar, in magnitude and direction of effects, to that found in models for trees (particularly deciduous), but fundamentally opposite compared to giant graminoids (Table 4). In short, aridity (low precipitation and high temperature), mediated or not by harshening of soil conditions (increasing EC, pH, ESP), seems to determine open canopies which in turn favor development of dense graminoid stands.

## Discussion

Our results on riparian vegetation support the view from other ecosystems that a PFTs’ approach can be a useful tool in modelling the effects of climate change on ecosystems (e.g., [23, 44, 45]). Congruent with our first hypothesis, PFTs showed general intra group cohesion when classified a posteriori by means of leaf traits related with leaf litter recalcitrance and nutritional value for invertebrate shredders. Our second hypothesis was generally supported, since changes in PFTs were predicted reasonably well from climate variables. However, our third hypothesis was poorly supported as, despite the notable predictive contribution from several soil variables, those were highly dependent on climate.

### Plant functional types and leaf traits related to leaf litter decomposability

In agreement with previous studies [15, 46], we found that PFTs of woody species were defined better by their leaf nutrient allocation strategies than by their growth forms. Deciduous and evergreen plants formed two distinct groups, regardless of whether they were trees or shrubs. Some studies have recommended caution when assigning average trait values to PFTs, as their overlap in trait ranges can be large [15, 16]. Our results suggest, however, that despite the relatively high intra-group dispersion in some trait data (e.g. SLA, toughness and C:N), and the lack of significant differences for lignin and Si between deciduous and evergreen plants, the first group showed significantly higher SLA and %N_mass_ but lower toughness and C:N ratio compared to the second. These two groups represent alternative life history strategies involving leaf traits associations (syndromes): i.e. trade-offs between the prevalence of high investment in photosynthesis and growth in deciduous species versus the preferential resource allocation to structural tissues to increase leaf lifespan and defense, and to reduce nutrient losses, of evergreen species [23, 47]. Giant graminoids showed notable leaf traits overlap with other groups, but were strikingly different from both evergreen and deciduous for their much lower lignin but higher Si content. This, again, reveals a fundamental dichotomy affecting C use, between the relatively high C investment of woody plants in leaf structure and defense (lignin) and the preferential C allocation to growth and reproduction while using silicon for structural and defense functions in graminoids [48].

Traits providing structure and protection to fresh leaves remain operational after dead [49, 50]. Fast decomposition rates have been associated to high SLA and N but low toughness and C:N ratio [51]. Further, low toughness has been demonstrated to be a good proxy of elevated nutritional value for invertebrate shredders [38]. Our deciduous and evergreen groups did not differ in lignin concentration, a major determinant of leaf litter decomposition in woody plants [51]; however, they significantly differed in most other leaf traits, so that we can expect higher decomposition rates and nutritional value for deciduous compared to evergreen. GG leaves were similarly tough as evergreen leaves, probably due to the high Si concentration in the first but due to low SLA and high fiber concentration in the second group. The accumulation of SiO_2_ promotes strengthening of the cell walls which, in turn, has been related to slow decomposition of leaf litter, both microbial-mediated [52, 53] and detritivore-mediated decomposition rates [54]. Thus, we could expect that environmental factors with detrimental effects on deciduous species or having a positive influence on evergreen plants or giant graminoids would have the potential to weaken terrestrial-aquatic linkages in headwater streams.

### Responses of plant functional types to environmental factors

Our results indicated a prime role of climate predicting changes in relative abundance and diversity of PFTs. In terrestrial ecosystems, for a long time, there was a general consensus on the preponderant effects of climate on leaf traits [55]; however recent studies state that climate, although being a significant predictor of PFTs fundamentally related with leaf traits, its predictive power alone is modest [16, 56], and even soil fertility effects can overwhelm those of climate [15, 57]. Studies in the Mediterranean region indicate that vegetation functional structure (based importantly on leaf traits) depends broadly on soil water availability [58] and its species richness on soil fertility [59]. These findings are in apparent contrast with our results, particularly if we consider the negligible role that climate-mediated soil water availability (via precipitation) should have on PFTs from permanent streams. Although, perhaps, our results might suggest a leading role of temperature—in the absence of major soil water limitation—ruling leaf physiology [60] and soil processes affecting plant performance (e.g. exacerbating salt accumulation near the soil surface as aridity increases, see below) with fundamental filtering effects, direct and indirect respectively, on leaf traits. Moreover, the relatively low predictive power of soil fertility variables—available nutrients or CEC—might be related to the high nutrient dynamics typical of alluvial soils, besides the fact that water is pivotal controlling nitrogen and phosphorus cycling in semiarid regions, regardless of local soil fertility [61]. Riparian soils are considered biogeochemical “hot spots”, i.e. terrestrial-aquatic interfaces where fluxes and transformations of nutrients are enhanced, sustaining elevated productivity compared to upland soils particularly in semiarid settings [62]. These properties could have blurred the response of PFTs to soil fertility, despite its large geological gradient studied.

Overall, models predicted diversity worse than relative abundance of PFTs. This could be related to the more complex interplay of factors determining diversity compared to abundance. Diversity in fluvial ecosystems is broadly mediated by climate factors, but also by the regime of hydrological disturbance interacting with the regional pool of colonists and local resource availability [63].

Giant graminoids were best predicted from climate and soil variables related with aridity. The accumulation of salt in the top layer of soils is common in wetlands from semiarid regions, where evaporation exceeds by far precipitation and leaching is insufficient to move salts out of the soil profile. These processes lead to elevated %BS and pH (>8.3) [64]. The streams in the arid extreme of our climate gradient are primarily groundwater—fed with infrequent and lagged responses to flooding, i.e. discrete springs from aquifers in calcareous or gypsum rocks [65]. Thus, the high weathering potential of these lithologies, scarce flooding that leads to reduced leaching, along with the fact that evaporation and concentration of salts often increase in discharge wetlands (versus recharge ones) [66], likely exacerbated soil salinity in the most arid riparian soils.

The most frequent or abundant giant graminoids (GG), common reed (*Phragmites australis* (Cav.) Steud. subsp. *australis*), giant reed (*Arundo donax* L.) and ravenna grass (*Tripidium ravennae* (L.) H. Scholz), are reported to be halotolerant [67, 68]. These species also thrive better under warm, high-irradiance climates [69], which is the common setting in our most arid, low-elevation streams, where tree canopy is absent or sparse. We found, indeed, that abundance and diversity of deciduous trees broadly showed a similar pattern of variation, but inverse, to that found for GG. This combination of competing patterns between PFTs seems to represents a vegetation syndrome tightly linked to aridity that has been already described in riparian ecosystems from SW North America. In permanent streams of mid-lowland rivers in this region has been reported a vast decline of broad leaves woody deciduous species concomitant with the expansion of the halotolerants common reed and giant reed [70] and/or the halophytic shrub *Tamarix* [71]. These changes have been associated with decreasing flood frequency and increasing soil salinity, along with the lower water use efficiency of woody deciduous compared to the invasive ones under these conditions [71].

Cover percentage of evergreen trees increased with increasing precipitation and moderation of moisture seasonality. This pattern can be likely related with a prominent weight on riparian canopy cover from big evergreen trees located at upland sites or riparian margins, in areas with relatively high annual rainfall (> 800 mm). Evergreeness is the common plant strategy in Mediterranean woodlands [72]. This influence from upland vegetation was negligible in streams from drier zones where scrublands or very open woodlands dominate. However, cover and diversity of evergreen shrubs (mostly arborescent broad leaves shrubs) and diversity of evergreen trees were also importantly favored by mild winters, besides increasing precipitation and moderate moisture seasonality. These conditions took place in relatively low elevation streams with maritime influence. Such a trend was roughly similar, but inverse, to that observed for deciduous trees, which were greatly favored by decreasing mean temperature with increasing altitude. The distribution of evergreen versus deciduous plants has been a subject of debate since it was first addressed by Monk in 1966 [47, 72]. One of the most widely-held interpretations is that the conservative evergreen strategy would be increasingly successful in relation to the more productive deciduous as the length and magnitude of the unfavorable season increases and/or soil fertility decreases [47]. Accordingly, the dominance of evergreen trees, compared to deciduous, in Mediterranean woodlands has often been explained by their capacity to cope better with constrains posed by the unfavorable summer-drought. This explanation, however, is untenable regarding relative abundance and diversity of evergreen shrubs in headwater streams. It is most likely that, in these ecosystems, constrains from low winter-temperature are to be of greater importance on plant performance than summer-drought. Winter cold stress, indeed, is one of the main factors limiting photochemical efficiency of evergreen species [73], which must impair this PFT notably at high altitudes and/or in continental areas of the Mediterranean region [74]. Thus, in permanent streams located in areas with mild winters, the conservative evergreen strategy would be remarkably successful, owing to their capacity of lengthening the growing season, offering a competitive advantage over winter deciduous. Results at the global scale, indicating that low thermal and moisture seasonality favors evergreen dominance [72], support this statement. Moreover, as most evergreen shrubs species recorded in our study area appear to have evolved from tropical-like environments during early Pliocene and pre-Pliocene (pre-Mediterranean lineages), it is likely that they would represent adaptations to paleoclimatic warm and humid conditions [75].

Without detracting from the key role of mild winters favoring evergreen shrubs, it is nonetheless true that high precipitation is of primary importance to explain abundance and diversity of this PFT. Perhaps this strong relationship could be associated to the capital effect of high rainfall, and associated increasing flooding frequency, to prevent salt accumulation in riparian soils, which would otherwise promotes the proliferation of giant graminoids under warm conditions.

### Implications for stream food web in a changing climate

Our relatively simple PFT classification appears useful to predict changes of PFTs in headwater streams as a result of changing climatic conditions, and their putative domino effects on stream food webs. Climate change projections for the Mediterranean region forecast pronounced warming and decrease in precipitation towards the end of the 21st century (see review in [2]). Within this scenario is to be expected a decline in the average streamflow, which may cause drying out of small streams [3]. But even if desiccation does not occur, our results suggest notable changes in stream food webs caused by aridification, due to a reduction in the more decomposable and palatable leaf litter inputs from deciduous vegetation and the concomitant increasing dominance of giant graminoids. While climate warming can be considered a very probable projection, precipitation projections are usually fraught with greater uncertainty. This is particularly true in the Mediterranean region where climate change signal shows substantial fine scale structure due to the complex topography and coastline of the region [2]. Thus, in a scenario of no precipitation reduction in presently wetter areas, the sole and highly probable projection of increasing mean temperature, heat waves during summer and decreased probability of cold events during winter [76], might foster the expansion of broad leaves evergreen to the detriment of deciduous species. The net balance of the former trends would be increasing leaf litter recalcitrance which might slow down the incorporation of energy and matter into detrital food webs. However, further research is needed to determine the magnitude of these changes.

## Supporting information

S1 Fig Study area (Andalusia, Southern Spain). Location of sampling sites (red dots) in protected areas, which are listed from 1 to 11. The color scale shows values of the UNEP index of aridity (annual precipitation / potential evapotranspiration). 1 to 4: areas under Mediterranean subdesert climate; 5 to 9, and 11: areas under Mediterranean inland and mountainous climate; 10: area under Mediterranean subtropical climate.
(DOCX)

S2 Fig Biplots of correlations of predictor (X) and dependent (Y) variables with latent variables (t1 and t2) extracted by multivariate dependent PLS regression. These multivariate models correspond to the sum of the environmental matrices (climate + soil + physical). For clarity, only are represented predictors with correlation ≥ 0.5. Dependent variables are % cover (a) and Simpson diversity (b) of the five plant functional types and for the whole riparian plant community. For interpretation of predictors acronyms see Table 1 or Materials and methods.
(DOCX)

S1 Table Type of riparian vegetation and species richness per site and plant functional type. The type of riparian vegetation is based on the EUNIS habitat classification* of the European Environment Agency (EEA) (http://eunis.eea.europa.eu/habitats.jsp): the first alpha-numeric code indicates the type of habitat according to this classification and the code in brackets refers to the habitat defined in the “EU Habitats Directive Annex I habitat types”. *The EUNIS habitat classification is a hierarchical pan-European system to facilitate the harmonised description and collection of data across Europe through the use of standard criteria.
(DOCX)

S1 Appendix Scientific name of species, family, plant functional type (PFT), and mean percent coverage recorded in the 34 streams studied. In bold, species which leaves have been collected and their traits analysed. PFT: deciduous shrubs (DS), deciduous trees (DT), evergreen shrubs (ES), evergreen trees (ET), giant graminoids (GG), and giant forbs (GF).
(XLSX)
