# Supplementary figures and images for: Climate-driven changes of riparian plant functional types in permanent headwater streams. Implications for stream food webs
Source: PLoS One. 2018 Jun 28;13(6):e0199898. doi: 10.1371/journal.pone.0199898 (PMC6023121; doi:10.1371/journal.pone.0199898)

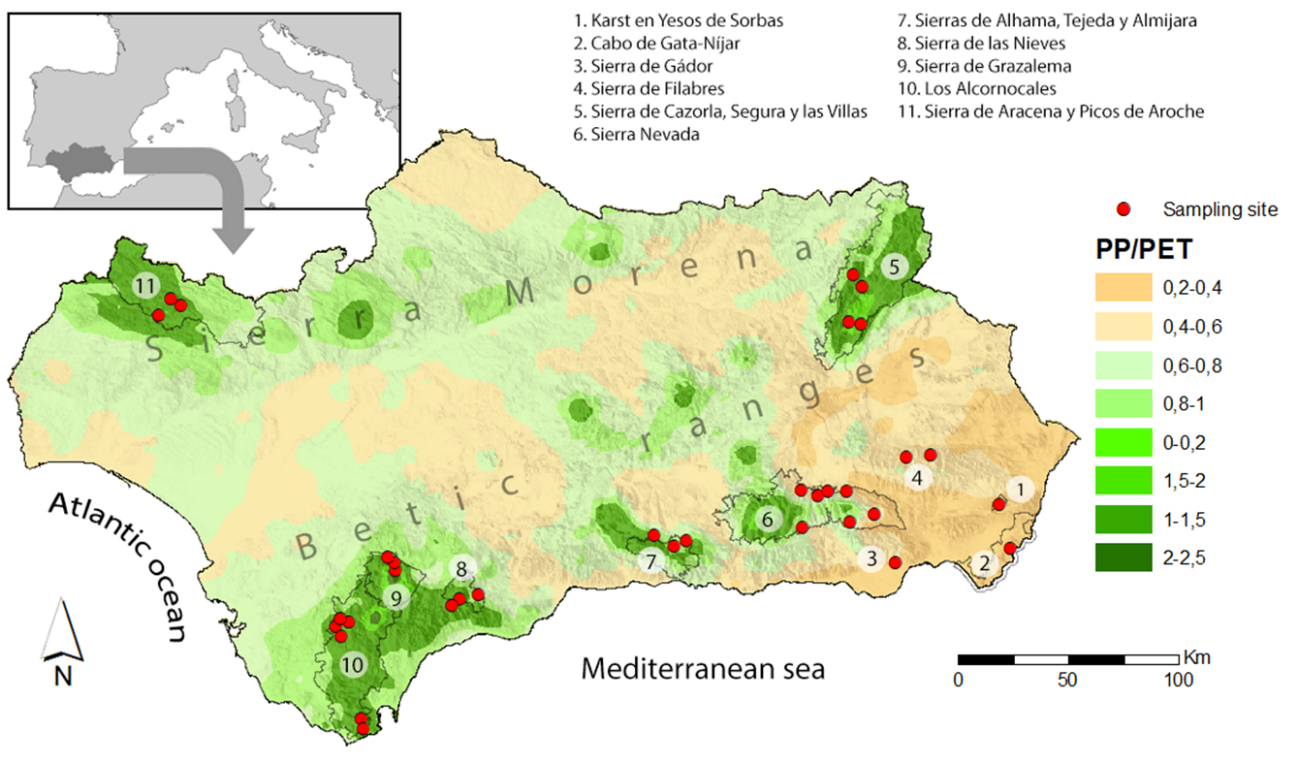

Supplement: S1 Fig — Location of sampling sites (red dots) in protected areas, which are listed from 1 to 11. The color scale shows values of the UNEP index of aridity (annual precipitation / potential evapotranspiration). 1 to 4: areas under Mediterranean subdesert climate; 5 to 9, and 11: areas under Mediterranean inland and mountainous climate; 10: area under Mediterranean subtropical climate. (DOCX) [file pone.0199898.s001.docx]

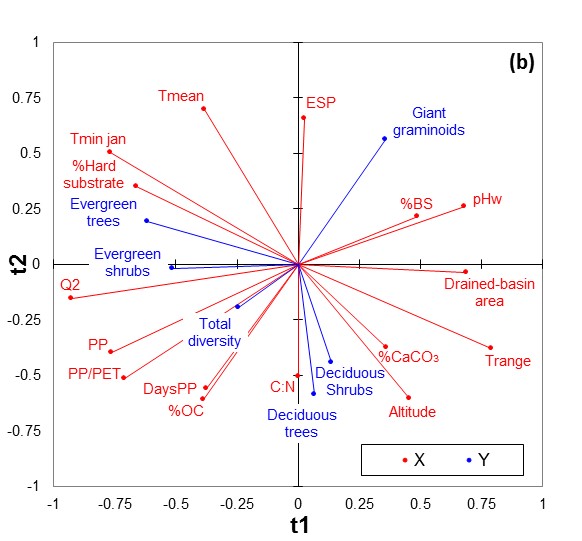

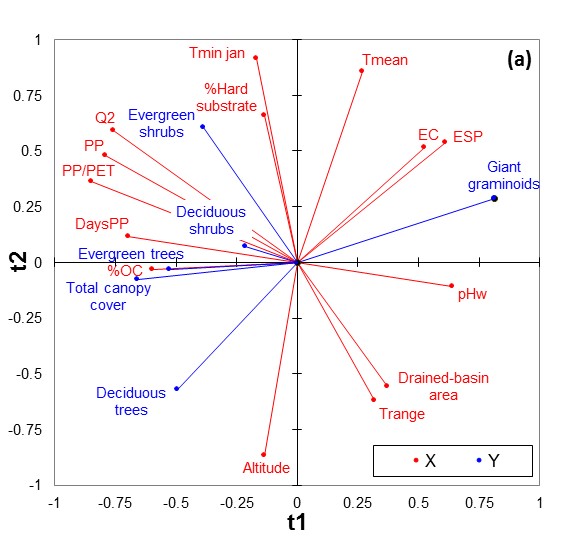

Supplement: S2 Fig — These multivariate models correspond to the sum of the environmental matrices (climate + soil + physical). For clarity, only are represented predictors with correlation ≥ 0.5. Dependent variables are % cover (a) and Simpson diversity (b) of the five plant functional types and for the whole riparian plant community. For interpretation of predictors acronyms see Table 1 or Materials and methods. (DOCX) [file pone.0199898.s002.docx]
