# Supplementary material for: Climate-driven changes of riparian plant functional types in permanent headwater streams. Implications for stream food webs
Source: PLoS One. 2018 Jun 28;13(6):e0199898. doi: 10.1371/journal.pone.0199898 (PMC6023121; doi:10.1371/journal.pone.0199898)
Supplement: S1 Table — The type of riparian vegetation is based on the EUNIS habitat classification* of the European Environment Agency (EEA) (http://eunis.eea.europa.eu/habitats.jsp): the first alpha-numeric code indicates the type of habitat according to this classification and the code in brackets refers to the habitat defined in the “EU Habitats Directive Annex I habitat types”. *The EUNIS habitat classification is a hierarchical pan-European system to facilitate the harmonised description and collection of data across Europe through the use of standard criteria. (DOCX) [file pone.0199898.s003.docx]

| Protected area | Stream | Latitude (N) | Longitude (W) | Type of riparian vegetation | Species richness | | | | | | |
| --- | --- | --- | --- | --- | --- | --- | --- | --- | --- | --- | --- |
|  |  |  |  |  | Deciduous shrubs | Deciduous trees | Evergreen shrubs | Evergreen trees | Giant graminoids | Giant forbs | Total |
| 1-Karst en Yesos de Sorbas | Río de Aguas-Molinos | 37°05'20.34" | 2°04'22.24" | G1.311. Iberian poplar galleries | 2 | 2 | 5 | 1 | 5 | 1 | 16 |
| 2-Cabo de Gata-Níjar | Arroyo de Las Negras | 36°53'32.72" | 2°00'39.03" | F9.311. Oleander galleries (92D0) | 2 | 0 | 14 | 0 | 5 | 0 | 21 |
| 3-Sierra de Gádor | Barranco del Cura | 36°50'06.57" | 2°38'18.78" | F9.311. Oleander galleries (92D0) | 1 | 1 | 11 | 2 | 3 | 1 | 19 |
| 4-Sierra de Filabres | Río Bacares | 37°18'18.88" | 2°26'40.95" | G1.311. Iberian poplar galleries | 6 | 7 | 4 | 0 | 1 | 1 | 19 |
|  | Arroyo de Los Marcos | 37°17'53.72" | 2°34'43.87" |  | 6 | 6 | 9 | 0 | 2 | 0 | 23 |
| 5-Sierras de Cazorla, Segura y las Villas | Arroyo de la Garganta | 37°53'47.20" | 2°53'37.81" | G1.1122. Andalusian olive-leaved willow woods | 4 | 4 | 4 | 2 | 1 | 0 | 15 |
|  | Río Guadalentín-Cortijo | 37°53'11.82" | 2°49'41.92" | G1.332. Iberian meso-Mediterranean ash galleries (91B0) | 4 | 9 | 5 | 1 | 1 | 1 | 21 |
|  | Río Aguascebas Grande | 38°06'07.06" | 2°51'59.75" | F9.124. Ibero-montane riverine willow scrub | 6 | 7 | 8 | 2 | 1 | 0 | 24 |
|  | Aguamulas | 38°03'06.63" | 2°49'05.89" | G1.311. Iberian poplar galleries | 7 | 5 | 12 | 1 | 2 | 0 | 27 |
| 6-Sierra Nevada | Río Andarax | 37°00'46.68" | 2°53'18.62" | G1.131. Southern *Alnus glutinosa* galleries | 2 | 5 | 1 | 2 | 0 | 0 | 10 |
|  | Río Chico-Ohanes | 37°02'51.78" | 2°45'25.28" | G1.1122. Andalusian olive-leaved willow woods | 1 | 9 | 7 | 0 | 0 | 0 | 17 |
|  | Río Mecina | 36°59'19.88" | 3°09'00.39" | G1.131. Southern *Alnus glutinosa* galleries | 3 | 5 | 2 | 1 | 1 | 2 | 14 |
|  | Río Nacimiento | 37°08'58.82" | 2°54'25.68" | G1.1122. Andalusian olive-leaved willow woods | 3 | 1 | 4 | 2 | 0 | 0 | 10 |
|  | Arroyo Hondo | 37°07'43.39" | 3°03'45.28" | G1.131. Southern *Alnus glutinosa* galleries | 3 | 7 | 1 | 1 | 0 | 1 | 13 |
|  | Arroyo de los Castaños | 37°09'03.07" | 3°00'30.30" | G1.131. Southern *Alnus glutinosa* galleries | 3 | 6 | 5 | 2 | 1 | 0 | 17 |
|  | Lanteira | 37°09'07.72" | 3°09'18.91" | G1.131. Southern *Alnus glutinosa* galleries | 5 | 7 | 3 | 2 | 0 | 0 | 17 |
| 7-Sierras de Alhama, Tejeda y Almijara | Río Alhama | 36°57'02.41" | 3°57'26.21" | G1.332. Iberian meso-Mediterranean ash galleries (91B0) | 5 | 6 | 0 | 0 | 2 | 2 | 15 |
|  | Río Vacal | 36°54'10.64" | 3°50'45.69" | G1.311. Iberian poplar galleries | 4 | 3 | 7 | 1 | 2 | 1 | 18 |
|  | Arroyo Turrillas | 36°55'46.56" | 3°46'51.57" | G1.1121. Mediterranean white willow galleries | 3 | 6 | 5 | 1 | 1 | 1 | 17 |
| 8-Sierra de las Nieves | Río Verde | 36°39'27.42" | 5°00'51.59" | F9.127. Pedicellated willow scrubs | 1 | 2 | 5 | 1 | 1 | 1 | 11 |
|  | Arroyo de los Caballos | 36°40'44.63" | 4°54'32.32" |  | 2 | 3 | 8 | 1 | 2 | 1 | 17 |
|  | Arroyo de la Cruz | 36°37'35.23" | 5°03'05.69" |  | 2 | 2 | 10 | 1 | 1 | 1 | 17 |
| 9-Sierra de Grazalema | Arroyo de Gaidóvar | 36°46'34.08" | 5°21'54.17" | G1.311. Iberian poplar galleries | 4 | 5 | 6 | 0 | 0 | 1 | 16 |
|  | Arroyomolinos | 36°48'38.64" | 5°22'25.38" | G1.311. Iberian poplar galleries | 3 | 4 | 11 | 2 | 1 | 0 | 21 |
|  | Río Bocaleones | 36°49'50.86" | 5°24'24.20" | F9.127. Pedicellated willow scrub | 4 | 4 | 11 | 2 | 2 | 0 | 23 |
| 10-Los Alcornocales | Garganta del Caballo | 36°31'24.96" | 5°40'58.11" | F9.1271. Andalusian willow shrub | 5 | 2 | 12 | 2 | 2 | 1 | 24 |
|  | Garganta del Aljibe | 36°32'39.99" | 5°36'33.45" | G1.132. *Rhododendron - Alnus* galleries (92B0) | 5 | 3 | 8 | 1 | 1 | 3 | 21 |
|  | Garganta de En medio | 36°33'20.37" | 5°39'30.41" | G1.132. *Rhododendron - Alnus* galleries (92B0) | 3 | 3 | 9 | 2 | 1 | 3 | 21 |
|  | Garganta de la Cierva | 36°28'46.79" | 5°39'02.85" | G1.132. *Rhododendron - Alnus* galleries (92B0) | 2 | 2 | 12 | 2 | 1 | 0 | 19 |
|  | Garganta de la Garza | 36°07'09.01" | 5°31'50.23" | G1.132. *Rhododendron - Alnus* galleries (92B0) | 4 | 3 | 10 | 3 | 0 | 3 | 23 |
|  | Garganta del Tesorillo | 36°04'27.44" | 5°31'06.48" | G1.132. *Rhododendron - Alnus* galleries (92B0) | 3 | 2 | 11 | 3 | 1 | 3 | 23 |
|  | Arroyo de Corterrangel | 37°54'51,61'' | 6°35'30,17'' | G1.131. Southern *Alnus glutinosa* galleries | 5 | 4 | 4 | 1 | 1 | 1 | 16 |
| 11-Sierra de Aracena y Picos de Aroche | Barranco Dundun | 37°56'23,05'' | 6°38'34,20'' |  | 6 | 2 | 7 | 2 | 1 | 3 | 21 |
|  | Ribera de Sta. Ana | 37°51'56,47'' | 6°42'16,82'' |  | 4 | 7 | 4 | 1 | 1 | 2 | 19 |
